# Geographic health inequalities in Norway: a Gini analysis of cross-county differences in mortality from 1980 to 2014

**DOI:** 10.1186/s12939-018-0771-7

**Published:** 2018-05-24

**Authors:** Eirin K. Skaftun, Stéphane Verguet, Ole F. Norheim, Kjell A. Johansson

**Affiliations:** 1 Department of Global Public Health and Primary Care, University of Bergen, Postboks 7804, N-5018 Bergen, Norway; 2 Department of Global Health and Population, Harvard T.H. Chan School of Public Health, Boston, MA USA; 3 Department of Drug and Addiction Medicine, Haukeland University Hospital, Bergen, Norway

**Keywords:** Inequality, Gini index, Mortality, Life expectancy, Norway

## Abstract

**Background:**

This study aims at quantifying the level and changes over time of inequality in age-specific mortality and life expectancy between the 19 Norwegian counties from 1980 to 2014.

**Methods:**

Data on population and mortality by county was obtained from Statistics Norway for 1980–2014. Life expectancy and age-specific mortality rates (0–4, 5–49 and 50–69 age groups) were estimated by year and county. Geographic inequality was described by the absolute Gini index annually.

**Results:**

Life expectancy in Norway has increased from 75.6 to 82.0 years, and the risk of death before the age of 70 has decreased from 26 to 14% from 1980 to 2014. The absolute Gini index decreased over the period 1980 to 2014 from 0.43 to 0.32 for life expectancy, from 0.012 to 0.0057 for the age group 50–69 years, from 0.0038 to 0.0022 for the age group 5–49 years, and from 0.0009 to 0.0006 for the age group 0–4 years. It will take between 2 and 32 years (national average 7 years) until the counties catch up with the life expectancy in the best performing county if their annual rates of increase remain unchanged.

**Conclusion:**

Using the absolute Gini index as a metric for monitoring changes in geographic inequality over time may be a valuable tool for informing public health policies. The absolute inequality in mortality and life expectancy between Norwegian counties has decreased from 1980 to 2014.

**Electronic supplementary material:**

The online version of this article (10.1186/s12939-018-0771-7) contains supplementary material, which is available to authorized users.

## Background

Geographical differences in life expectancy and mortality has been shown to be present within several countries [1–4]. Even if equality in health is an important political goal, countries rarely systematically report the size of inequality in survival across geographical areas. A numerical distributive metric needs to be implemented if the performance on geographical inequality is to be tracked and used to inform policies. In this paper, we will show how one such method can be used on Norwegian data.

The causes of geographic inequality are complex and only partly known, and may be amenable to interventions both within and outside the health sector. Constant or increasing geographic inequality in life expectancy have been documented in New Zealand and the European Union [5, 6]. Regional differences in treatment and survival for severe diseases have been identified in several countries [7–9]. Indicators of socioeconomic status, such as low levels of income or education, and health behaviour, such as smoking and exercise, have also been shown to be associated with geographic inequalities [2, 10–12]. Studies on how travel time to the nearest hospital affect mortality yield more mixed results [7, 13, 14].

Over the past 50 years, Norway has experienced economic growth and substantial improvements in welfare and population health. The total population of Norway is around 5.2 million and the country is administratively divided into 19 counties, with populations ranging from 75,000 in the county Finnmark located in the north to 648,000 in the capital city and county of Oslo as per January 1st 2015 [15]. County population density ranges from 1500 inhabitants per square kilometer in Oslo to two inhabitants per square kilometer in Finnmark [16]. The Norwegian health system is semi-centralized and mainly publically financed. Hospitals and specialized health services were provided by the counties until the state took over this responsibility in 2002. Hospitals are located in all counties, but the patients’ travel time varies. In 2010, 45% of the population in the three counties in northern Norway had a travel time exceeding one hour to the nearest hospital with an emergency obstetric department, while this proportion was well below 10% in the more densely populated counties in the south and east [13]. The responsibility for primary health care lies with the municipalities. A diverse geography entails local differences in industry, economic activity, culture, and life style, and socio-economic indicators and health-related lifestyle factors differ between counties [17, 18].

Despite political interest in health inequalities, there is no systematic monitoring of geographic inequalities in health. Systematic monitoring of these inequalities will provide relevant information for policymakers concerned with reducing the inequalities. This study aims to provide an empirical illustration of how geographic inequalities can be monitored using the Gini index by applying this metric to Norwegian mortality data from the past 35 years. Additionally, this study explores a didactic way of presenting the magnitude of inequality.

## Methods

### Data and estimation of life tables

Data on deaths and population as of January 1st for males and females was purchased from Statistics Norway for the period 1980–2014 [15]. Data on deaths and population was age-specific with the following age intervals: 0–1 years, 1–4 years, and five-year age group from age 5 to age 95 (0, 1–4, 5–9, 10–14, …, 85–89, 90–94, 95 years and above). We estimated full life tables for men and women for Norway and the 19 Norwegian counties, using standard life table methodology. Mean population for each year was estimated as the population in that year plus the population of the subsequent year, divided by two. For 2014 we used the population from January 1st 2014 due to data limitations. The intensity of death was estimated as the number of deaths divided by the mean population. We assumed deaths to be evenly distributed across the time interval age *x* to age *x + 1* years, with exception of the age group 0–1 years where we estimated the distribution of the deaths according to the methodology described in the Human Mortality Database’s Methods Protocol [19]. Life tables were computed for one-year periods and for five-year periods (1980–1984, 1985–1989, ..., 2010–2014), for males and females separately and for both sexes combined. Life expectancy at birth and risk of dying for the age groups 0–4 years, 5–49 years, 50–69 years, and 0–69 years were extracted from the life tables.

Estimation of life tables and statistical analysis were done using STATA IC version 12.0 [20]. ArcMap was used for graphical visualization of the life expectancy for each five year period [21].

### Geographic inequality

Geographic inequality in risks of death and life expectancy at birth between the counties was measured for each year by the absolute Gini index. This is a comprehensive measure of inequality encompassing the distribution of inequality in each of the counties, and is interpreted as half the average difference between any two counties [22]. For example, if the absolute Gini index for life expectancy is 0.3, this would mean that the average difference in life expectancy between two randomly selected counties is 0.6 years.

The Gini index is closely related to the Lorenz curve, which plots the cumulative proportion of the outcome variable against the cumulative proportion of people ranked by the outcome [23, 24]. The absolute Gini index is defined as twice the area between the Lorenz curve and the diagonal line, multiplied by the mean value of the variable of interest. For each of the variables (age-specific risk of death and life expectancy at birth), the absolute Gini index was calculated for each year based on each county ranked from worst to best level of the variable, and weighed by the size of the population in the corresponding age, using the following formula [25]:

$$ G=2\sum \limits_{t-1}^T{\mu}_t\times {f}_t\times {R}_t-\mu, $$

where G is the absolute Gini index, μ is the mean value of the variable, T the number of counties, μ_t_ the value of the variable in the t^th^ county, f_t_ the county’s population share, and R_t_ the relative rank of the t^th^ county. For graphical presentation of the trends over time, fitted curves using third degree polynomial regression were constructed according to the following formula:

$$ \mathrm{f}(x)=\mathrm{a}{x}^3+\mathrm{b}{x}^2+\mathrm{c}x+\mathrm{d}. $$

Where χ is the year, f(χ) the estimated value of the Gini index in year χ, and a, b, c and d the constants determined by the regression model.

### Rates of change

The rates of change have been estimated to provide a didactic illustration of the magnitude of the inequalities. Even if the Gini index contains many appealing theoretical equity concerns and is a precise distributive metric, such an index may be difficult to interpret for many. As an addition, building on a previously developed model [26], we therefore calculate how many years it would take for each county to reach the best performing county. This is meant as an illustration of the gap between best and worst performers. The average annual rate of change over the period 2005–2014 was estimated as the first derivative of life expectancy at birth or risk of death by year for males and females combined. The best performing county in 2014 was identified, i.e. the county with the lowest risk of death or the highest life expectancy. The average annual rates of change were further applied to each county’s risk of death and life expectancy in 2014 in two different manners. First, we applied, for each county, the county’s own rate of change to the county’s risk of death and life expectancy in 2014. We computed how many years it would take to reach the level of life expectancy or risk of death found in the best performing county in 2014, assuming the annual rate of change to be unchanged. Second, we applied the best rate of change found in any county to the 2014-level in all counties, and estimated how many years it would take for each county to reach the 2014-level of the best performing county.

## Results

**Fig. 1:**
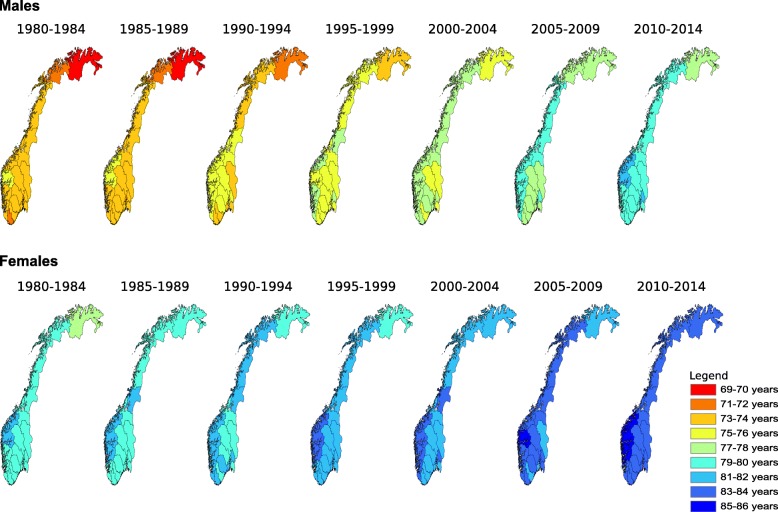
Life expectancy at birth in Norway by county (1980–2014). The maps are based on life tables estimated for five-year periods

Life expectancy at birth in Norway increased in all counties from 1980 to 2014. The increase over this period was from 72.3 to 79.9 years for males, from 79.0 to 84.0 years for females. The absolute difference in life expectancy at birth between the county with the highest and lowest life expectancy has decreased from 5.2 to 4.6 years for males, and increased from 3.4 to 3.9 years for females from 1980 to 2014. Figure 1 displays the change in life expectancy at birth from 1980 to 2014 for males and females separately by county.

**Fig. 2:**
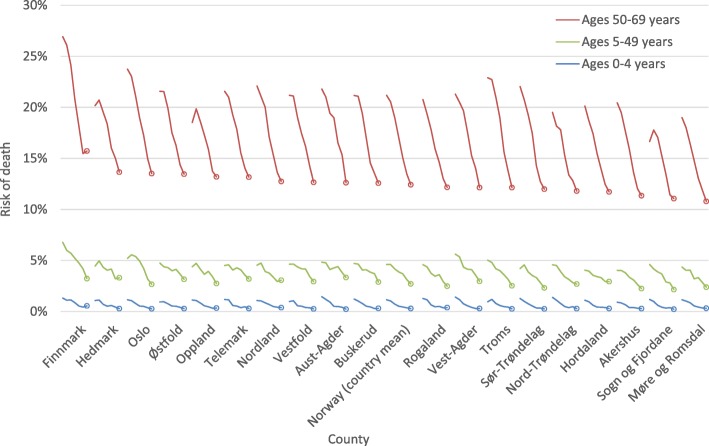
Trendlines for risks of death in selected age ranges, 1980–2014. Risk of death at ages 0–4, 5–49 and 50–69, based on life tables estimated for five-year periods. The circle represents the risk of death for the period 2010–2014

Figure 2 shows the change over time in risks of death by county for the age groups 0–4 years, 5–49 years and 50–69 years for both sexes combined, sex-specific results can be found in the Additional file 1: Risk of death by county, age group and sex. All the age-specific risks of death are decreasing in all counties, and the risk of death before age 70 has been reduced from 26 to 14% in the period 1980–2014. Some counties stand out as best and worst performers in terms of risk of death before age 70. Finnmark has the highest risk of death before age 70 in 28 out of the 35 years. The counties Sogn og Fjordane and Møre og Romsdal, both located in the western part of Norway, have the lowest risk of deaths before age 70 in 14 and 12 out of the 35 years, respectively.

**Fig. 3:**
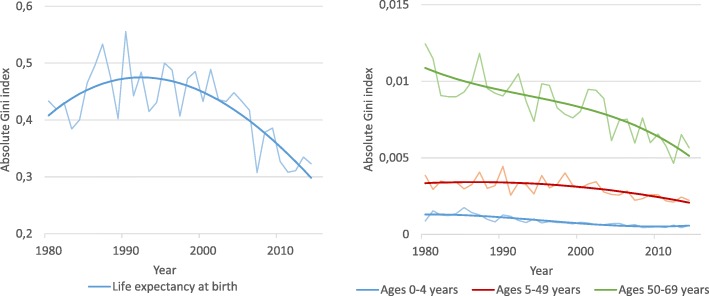
Geographic inequalities in risks of death at ages 0–4, 5–49 and 50–69 and life expectancy at birth, 1980–2014. Trendlines are smoothed using third degree polynomial regression

The geographic inequality, as measured by the absolute Gini index, is graphically shown for life expectancy and age-specific risks of death in Fig. 3. Sex-specific results can be found in the Additional file 2: Absolute Gini index by sex. Over the period 1980–2014, the absolute Gini index decreased for all age-specific mortality rates. The Gini index decreased from 0.0120 to 0.0057 for the age group 50–69 years, from 0.0038 to 0.0022 for the age group 5–49 years, and from 0.0009 to 0.0006 for the age group 0–4 years. The absolute Gini index for life expectancy at birth increased from 0.43 in 1980 and reached a maximum of 0.55 in the early 1990s. From the early 1990s to 2014, the absolute Gini index for life expectancy decreased to 0.32.

The average annual rates of change over the past ten years are shown in Table 1. The table also presents estimates of how many years it will take for each county to catch up with the 2014-level of the best performing county under different scenarios. For life expectancy, the best performing county in 2014 was Sogn og Fjordane with a life expectancy at birth of 83.1 years. In the first scenario, all the other counties increase life expectancy at the same rate as they have done over the past 10 years. It would then take between 2 and 32 years (national average 7 years) until the other counties reach the life expectancy that Sogn og Fjordane had in 2014. In the second scenario, the increase in life expectancy in each county equals the highest increase found across all counties. Under this scenario, it would take between 1 and 14 years (national average 5 years) until the other counties reaches the life expectancy of Sogn og Fjordane in 2014.

**Table 1:** Annual rates of change 2005–2014 and years until each county reaches the 2014-level of the best performing county

	Risk of death ages 0–4 years (5q0)			Risk of death ages 5–49 years (45q5)			Risk of death ages 50–69 years (20q50)			Life expectancy		
	Yearly rate of change (in 10^− 4^)	Years to catch-up^a^	Years to catch-up^b^	Yearly rate of change (in 10^− 4^)	Years to catch-up^a^	Years to catch-up^b^	Yearly rate of change (in 10^− 4^)	Years to catch-up^a^	Years to catch-up^b^	Yearly rate of change (in 10^−4^)	Years to catch-up^a^	Years to catch-up^b^
Aust-Agder	−3.50	5	5	−6.91	30	11	−52.19	7	7	0.27	9	8
Akershus	−0.57	44	6	−8.76	7	3	−15.63	7	3	0.17	4	3
Buskerud	−1.07	Ref.^c^	Ref.^c^	−14.46	11	9	−17.98	16	6	0.25	5	5
Finnmark	1.90	N/A^d^	13	−19.27	9	9	−3.65	167	12	0.12	32	14
Hedmark	−2.24	4	3	0.47	N/A^d^	8	−23.83	17	8	0.16	14	8
Hordaland	−1.81	18	8	−4.22	29	7	−12.67	15	4	0.13	7	3
Møre og Romsdal	−0.63	40	6	−9.65	5	3	−21.16	Ref.^c^	Ref.^c^	0.17	2	1
Nordland	−0.11	336	9	2.07	N/A^d^	6	−22.74	10	5	0.12	12	5
Nord-Trøndelag	−2.49	6	4	−3.83	21	5	−13.29	19	5	0.16	9	6
Oppland	0.90	N/A^d^	9	−11.87	12	8	−16.44	18	6	0.14	16	8
Oslo	−1.92	12	6	−10.70	9	5	−27.49	12	7	0.28	4	4
Østfold	−2.16	5	3	−8.64	16	8	−22.38	15	7	0.18	12	8
Rogaland	0.02	N/A^d^	7	−9.43	6	3	−23.73	10	5	0.16	4	3
Sogn og Fjordane	−2.52	1	1	−13.25	Ref.^c^	Ref.^c^	−3.92	39	3	0.16	Ref.^c^	Ref.^c^
Sør-Trøndelag	−1.11	6	2	−8.00	12	5	−17.09	14	5	0.21	5	4
Telemark	−3.88	6	6	−9.49	11	6	−10.23	30	6	0.20	9	7
Troms	−4.19	2	2	−13.13	6	5	−31.41	5	3	0.28	5	5
Vest-Agder	−0.10	350	9	−12.18	8	5	−38.33	4	3	0.23	3	3
Vestfold	−1.34	19	6	−11.05	13	8	−29.07	8	4	0.23	7	6
Norway	−1.31	17	6	−8.82	12	6	−21.18	11	5	0.19	7	5

^a^Years until the county reaches the level of the best performing county in 2014 if the counties’ yearly rates of change remain unchanged

^b^Years until the county reaches the level of the best performing county in 2014 if the yearly rate of reduction is equal to the best performing county’s rate of reduction

^c^The reference county is the best performing county in 2014

^d^N/A: Not applicable because of positive rate of change

## Discussion

Life expectancy varied substantially between counties, with a gap of 4.6 years for men and 3.9 years for women in 2014. Life expectancy and risks of death improved in all Norwegian counties from 1980 to 2014. The geographic inequality decreased; the absolute Gini index decreased from 0.43 in 1980 to 0.32 in 2014 for life expectancy and from 0.012 in 1980 to 0.0057 in 2014 for age specific risks of death in the age group 50–69 years.

The magnitude of inequality and the change in inequality over time differ according to the outcome measure used. The value of the absolute Gini index relates directly to the indicator that is measured, it is therefore not meaningful to compare the magnitude of an absolute Gini index for life expectancy with the absolute Gini index for a mortality rate, but the trends can be assessed over time for each of the indicators separately. An absolute Gini index of 0.32 for life expectancy in 2014 means that the average difference between the life expectancy in two randomly chosen counties is twice the value of this estimate, namely 0.64 years. The absolute inequality in mortality is decreasing or remaining unchanged for all age groups. For life expectancy, the trend shows an increase in inequality from 1980, peaking in the early 1990s and then decreasing again to below 1980 levels. The shape of the trend line should be interpreted with caution, as the shape is influenced by the method for smoothing the graph, and the lack of data before 1980.

Assessing whether the situation is getting more or less equal rely on several normative and technical choices underlying any measurement of inequality, and it is therefore important to understand the assumptions underlying the metric used [27]. The Gini index is a metric that incorporates the performance of all counties, and is thus more comprehensive than comparing the gap between best and worst performer or measures of how each county perform compared to e.g. the national average. Other comprehensive measures of inequality, such as the Atkinson index, the index of dissimilarity or the relative index of inequality, could have been used, advantages and disadvantages of different measures of inequality has been discussed in detail elsewhere [25, 28–30].

In this study an absolute measure of inequality is used, but it is not an obvious principled difference between a relative and absolute Gini measure of inequality [31]. Nevertheless, empirical evidence suggests that declining mortality in all groups is often associated with increasing relative and decreasing absolute inequality in mortality, and it has therefore been argued that reducing absolute inequality should be the main objective of public health policies [32, 33]. The Gini index incorporates a certain degree of inequality aversion. The inequality aversion parameter in the standard Gini index is two, leading to respective weights of 2, 1.5, 1, 0.5 and 0 for the health of the 0th, 25th, 50th, 75th and 100th percentile of the counties ranked by health, as shown by Wagstaff [34]. This parameter can be changed to allow for a different degree of inequality aversion.

The 19 Norwegian counties were chosen as geographical units for the analyses. Even though there are differences also within the counties, the counties differ from each other by population size and density, degree of urbanization, infrastructure, physical environment, main industries, and socio-economic characteristics of the population. Counties are especially relevant when it comes to the health sector as the responsibility for hospitals lied with the counties until 2002. Even with changing organizational structures, hospital services still follow county boundaries to a large degree. However, other geographical units could have been used for the study. The five health regions created in 2002 to manage secondary and tertiary levels of health care (reduced to four regions in 2007) would be a relevant geographical unit for linking the health inequalities to current and future policies governing specialized health care, but at the same time, important information would be lost if analyzing only 4–5 regions. The approximately 420 Norwegian municipalities could also have been used as units for the analysis, but estimates of life expectancy have been shown to be very uncertain if estimated for smaller administrative units than counties due to small populations [35]. Another option would be urban areas, but then rural populations would not be included in the analysis. Counties were therefore preferred as geographical units for the analysis.

This study adds empirical evidence at a macro level to ethical and political discussions. The inequalities quantified in this study encompass both current disparities in education, income, smoking etc., and measures aiming to compensate the effect of inequality in these underlying factors. Forces driving inequality and forces reducing inequality exist and interact in complex patterns, and it is useful for policy makers to monitor the overall effect of these factors on inequality. Information on the absolute level of inequality between counties regardless of the individuals’ socioeconomic position is therefore a useful supplement to information on specific causal pathways or subgroups of the population.

There are two principal explanations of the observed associations between place of residence and mortality. First, the individual characteristics of the people living in the area, such as the individuals’ education and income, may lead to differences in mortality (compositional factors). Second, characteristics of the area itself, such as infrastructure and availability of health services, may influence the individuals’ health and health-related behaviour (contextual factors). Regional inequalities in mortality are found to persist in Norway after adjustments for factors such as income, education and marital status in other studies [2, 36]. Effects of contextual factors and unmeasured effects of compositional factors are two possible explanations of the inequality that remains after adjusting for socioeconomic factors. Socioeconomic status is associated with mortality in various contexts and can be understood as a fundamental cause of inequality that influences a large number of causal pathways [37, 38]. Geographic inequalities have been found to be larger for the subgroups of the population with lowest socioeconomic status compared to the more affluent groups of society in several countries [2, 12]. Potential explanations for such findings include differences in the composition of the low-income groups, beneficial effects of living in an area with high average socioeconomic status, and differences in public policies and services. We did not assess the relative contribution of contextual and compositional factors to overall geographic inequality due to data limitations. This is an important limitation of our study. Further research is needed to assess the extent to which the geographic inequality is due to compositional and contextual factors, and to identify possible interventions that can enhance the positive development seen in Norway since 1980.

Measuring the number of years between the points in time when the counties will have the same level of life expectancy or age-specific mortality is a way of illustrating what the inequality means [26, 39]. Even though the inequality, as measured by the Gini index, is quite small for life expectancy and age-specific risks of death, it will still take many years for the worse-off counties to catch up with the best performing counties if the annual rates of change remain unchanged. For example, Finnmark is the county with the highest mortality in the age group 50–69 years and at the same time the county where improvements are happening at the slowest pace. If improvements continue to happen at the same pace as today, Finnmark will need 167 years to catch up with the best performer. If Finnmark managed to accelerate the progress and reduce mortality at the same pace as Aust-Agder, Finnmark would only need 12 years to reach the mortality risk seen in the best performing county. Identifying the counties where rapid progress has been made should be further explored in order to identify policies or contextual changes that may have contributed to the rapid improvement. Some of these factors, such as locally implemented public health policies, may be transferable to other counties and help policy makers in other places to accelerate progress.

## Conclusion

Life expectancy in Norway has increased in all counties and risk of death before the age of 70 years has been almost halved from 1980 to 2014. Although there is a political interest in geographic health inequality, there is no systematic monitoring in place in Norway. This study shows how an absolute geographic Gini index can be used for this purpose, and explores the changes in inequality between Norwegian counties over the past 35 years. Absolute inequality has decreased both for age-specific mortality and life expectancy at birth. Even with relatively low levels of geographic inequality, it may take many years for some counties to catch up with the best performing counties, if the yearly rates of improvement remain unchanged.

## Additional files

Additional file 1: Trendlines for risks of death in each county for selected age ranges, 1980–2014. (PDF 538 kb)

Additional file 2: Absolute Gini indices for life expectancy at birth and risks of death at ages 0–4, 5–49 and 50–69, 1980–2014. (PDF 330 kb)
