# Supplementary material for: Geographic health inequalities in Norway: a Gini analysis of cross-county differences in mortality from 1980 to 2014
Source: Int J Equity Health. 2018 May 24;17:64. doi: 10.1186/s12939-018-0771-7 (PMC5968669; doi:10.1186/s12939-018-0771-7)
Supplement: Supplementary file 1 — Trendlines for risks of death in each county for selected age ranges, 1980–2014. (PDF 538 kb) [file 12939_2018_771_MOESM1_ESM.pdf]

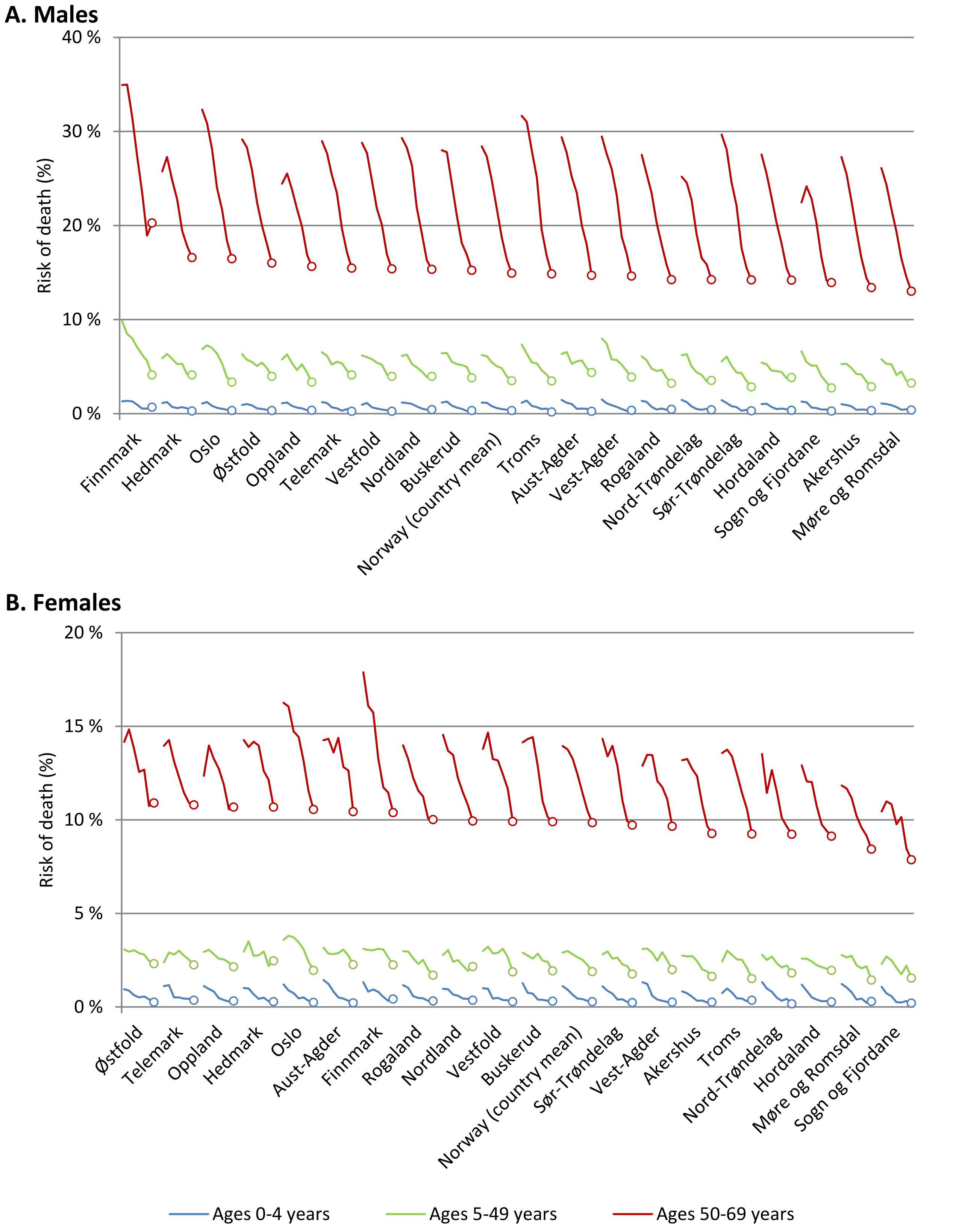

Appendix 1. Trendlines for risks of death in each county for selected age ranges, 1980-2014. The graphs are based on life tables estimated for five year periods. The circle represents the risk of death for the period 2010-2014. A: Males. B: Females.
