# Supplementary material for: Geographic health inequalities in Norway: a Gini analysis of cross-county differences in mortality from 1980 to 2014
Source: Int J Equity Health. 2018 May 24;17:64. doi: 10.1186/s12939-018-0771-7 (PMC5968669; doi:10.1186/s12939-018-0771-7)
Supplement: Supplementary file 2 — Absolute Gini indices for life expectancy at birth and risks of death at ages 0–4, 5–49 and 50–69, 1980–2014. (PDF 330 kb) [file 12939_2018_771_MOESM2_ESM.pdf]

A. Females

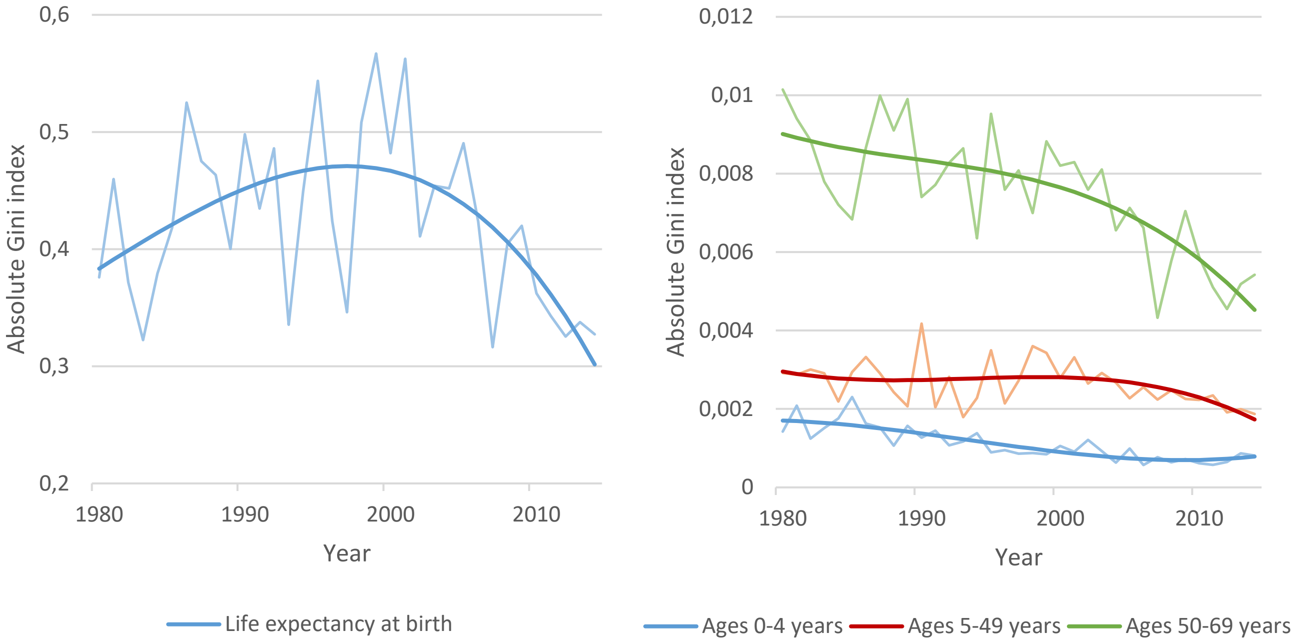

B. Males

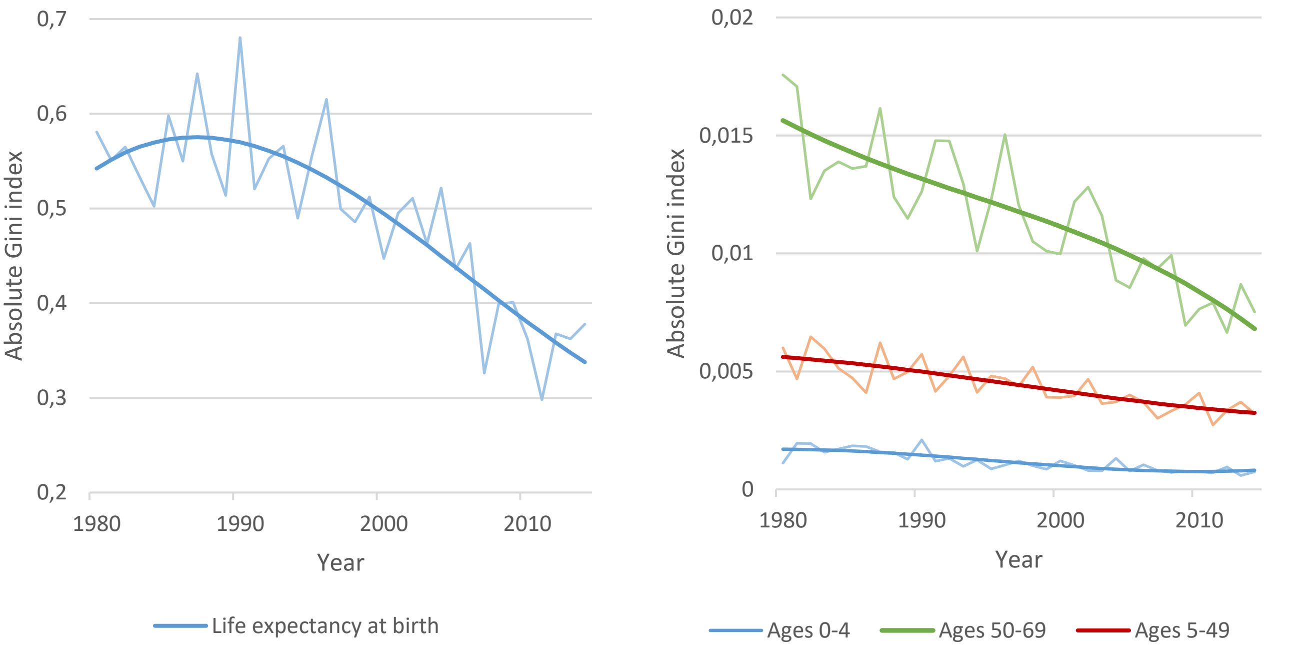

Appendix 2. Absolute Gini indices for life expectancy at birth and risks of death at ages 0-4, 5-49 and 50-69, 1980-2014. All trendlines are smoothed using third degree regression. A: Females. B: Males.
